# Failure of conventional retrograde cystography to detect bladder ruptures in pelvic trauma

**DOI:** 10.1007/s10195-010-0123-x

**Published:** 2010-12-08

**Authors:** O. Berber, C. Emeagi, M. Perry, M. S. Rickman

**Affiliations:** 1 Department of Trauma and Orthopaedics, St Georges Healthcare NHS Trust, Blackshaw Road, Tooting, London, SW17 0QT UK; 2 Department of Urological Surgery, St Georges Healthcare NHS Trust, London, UK

**Keywords:** Pelvic trauma, Bladder rupture, Cystography

## Abstract

Conventional retrograde cystography is often used to investigate patients with suspected bladder ruptures in pelvic trauma. Clinical indicators suggestive of a rupture include haematuria and suprapubic tenderness and should increase the suspicion of bladder and urinary tract injury and prompt the clinician to undertake further investigations. Two patients with high-energy pelvic fractures had bladder ruptures detected intraoperatively despite normal preoperative retrograde cystogram. Both patients had significant clinical indicators suggestive of underlying bladder and urinary tract injury. In both cases, a routine conventional retrograde cystogram was performed but failed to identify the full extent of the bladder injury. A possible reason for misdiagnosis in these cases is the delay between injury and investigation due to tertiary referral of care.

## Introduction

Bladder injuries occur in up to 8% of cases with pelvic trauma [1]. Many clinical indicators are suggestive and should prompt the clinician to undertake further investigations. Mortality rates range from 11% to 44% [2], but this is commonly attributed to the associated polytrauma [3]. Conventional retrograde cystography has been considered the gold standard for investigating such patients [1, 4] and has a reported sensitivity of 100% [1, 3–6]. More recently, computed tomography (CT) cystography has been used as an adjunct to trauma CT to evaluate bladder trauma, showing similar accuracy rates [6]. This procedure involves retrograde administration of contrast into the bladder through a catheter and performing a further scan in addition to the trauma CT. [6]. We describe two cases in which conventional retrograde cystography failed to detect a bladder rupture in patients with an associated pelvic fracture. In both cases, informed consent was obtained for inclusion in this report. The purpose of this case report is to highlight such occurrences, the possible reason for failure and the implications occurring as a result.

## Case reports

### Case 1

**Fig. 1:**
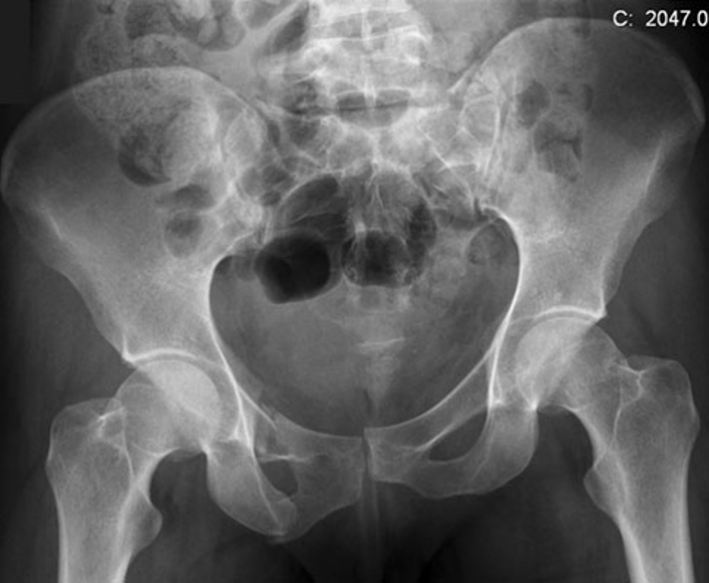
Initial pelvic injuries seen on an anteroposterior pelvic radiograph for case 1. The pelvic injury comprised right superior and inferior rami fractures with an associated pubic symphysis diastasis and a left sacral fracture

A 26-year-old woman sustained multiple injuries, including a pelvic fracture, after falling 20 feet (Fig. 1). The patient was transferred to a trauma centre for primary treatment. Initial assessment revealed macroscopic haematuria. A CT scan did not demonstrate contrast extravasation from the bladder. Urologists were consulted, and as the degree haematuria was settling, conservative treatment was chosen. Subsequently, the patient was transferred to a regional pelvic trauma unit for further treatment.

**Fig. 2:**
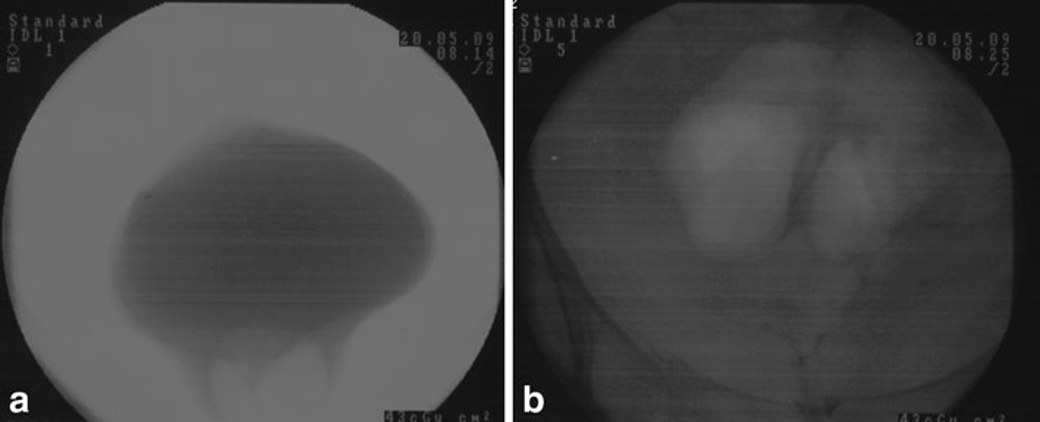
Retrograde cystography in case 1 demonstrating full (**a**) and postdrainage (**b**) films in assessing bladder injury in case 1. No leak was identified using 350 ml of contrast

**Fig. 3:**
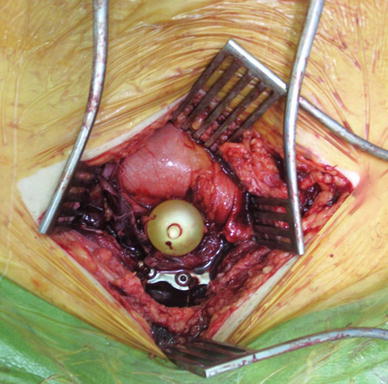
Intraoperative image in case 1 demonstrating the bladder rupture through a Pfannenstiel incision. The rupture was approximately 5 cm long extending from behind the pubic symphysis. Balloon of the urethral catheter is visible inside the bladder. Note the pubic symphysis diastasis plate in situ

At 7 days following injury, a retrograde cystogram was performed prior to definitive pelvic fracture stabilisation, which is routine practice at the hospital. The cystogram was performed using 250 ml of iodinated contrast diluted with 100 ml of normal saline. No leak was identified on full and postdrainage bladder films (Fig. 2a, b). A Pfannenstiel incision was then used to approach the pelvic fractures. On dividing the rectus muscle, significant amounts of clear fluid were encountered, raising the suspicion of bladder injury. Direct visualisation of the bladder revealed a tear approximating 5 cm and extending from behind the pubic symphysis (Fig. 3). The edges were rounded and fibrous, and much of the bladder wall was adherent to the surrounding tissues.

After copious irrigation, the fractures were stabilised as previously planned, and the bladder was repaired by a urologist. Urethral and suprapubic catheters were left in situ. Follow-up urethrocystograms revealed no remaining leaks, and catheters were sequentially removed.

### Case 2

**Fig. 4:**
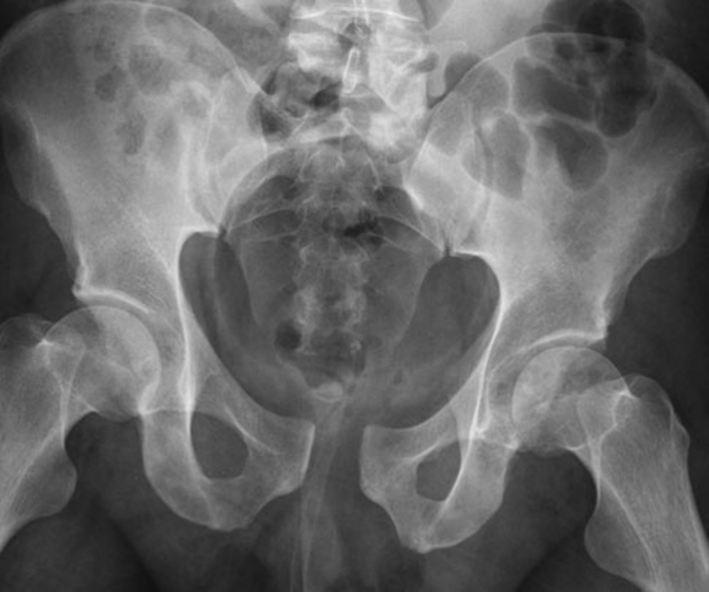
Initial pelvic injuries seen on an anteroposterior pelvic radiograph in case 2 include pubic symphysis diastasis, right sacroiliac diastasis and right sacral ala fracture

A 36-year-old man was involved in a 45-mph car accident. Multiple injuries were sustained, including a lateral compression-type pelvic injury (Fig. 4) and a perineal laceration.

After initial trauma surveys, he was catheterised without difficulty and had haematuria. A urology review was undertaken and a decision made to manage conservatively. A contrast-enhanced CT scan did not identify kidney or bladder injury. The patient was transferred to a regional pelvic trauma unit for pelvic stabilisation 10 days after the initial injury. A routine preoperative urethrocystogram revealed a small bladder-neck tear. Urology input was sought to confirm this, and a urethral catheter was left in situ. A laparotomy was performed to create a defunctioning colostomy. On entering the peritoneal cavity, further intraperitoneal and extraperitoneal ruptures were seen in addition to the bladder-neck tear. Both injuries were repaired by urologists and a suprapubic catheter inserted. An external fixator was applied to stabilise the pelvis. A urethrocystogram 4 weeks after bladder repair revealed no further leaks, and catheters were sequentially removed.

## Discussion

Bladder ruptures can be categorised into extraperitoneal (60%), intraperitoneal (30%) and combined (10–12%) [7]. Extraperitoneal ruptures are usually associated with pelvic fractures, most commonly occurring along the anterolateral aspect or bladder base. These injuries are due either to a shearing effect with disruption of the pelvis or direct bony penetration [3]. Intraperitoneal ruptures result from severe blunt lower-abdominal or pelvic trauma to a distended bladder. This leads to sudden intravesical pressure increase and rupture, often creating horizontal tears at the dome being the weakest point. Lateral compression fractures and pubic symphysis diastasis injuries are more commonly associated with bladder injuries [1]. Despite occurring relatively commonly, bladder injuries are still missed. In a recent study, 1% of cases were missed due to misinterpretation of cystogram images [8]. The clinical indicators suggestive of bladder trauma include suprapubic pain or tenderness, inability to void and low urine output. Unfortunately, in the presence of a pelvic fracture, these signs are almost always present.

The investigation of choice in our institution for suspected bladder injuries is retrograde cystography. Increasingly, retrograde CT cystography is being used with similar accuracy rates [1, 3–6]. To prevent false negatives, the bladder should be adequately distended with at least 250–300ml of fluid; radiographs should be taken in at least two projections and include a postdrainage film to visualise extravasated contrast [9]. No postdrainage films are needed for CT cystography.

In case 2 in this report, a correctly identified simple bladder-neck tear could have been treated with a urethral catheter alone. However, the cystogram failed to detect additional intraperitoneal and extraperitoneal ruptures, which represent more significant injuries requiring formal repair and a suprapubic catheter. One of the possible reasons the cystogram failed to detect the full extent of the leaks is the time span from injury to investigation—7 days in case 1 and 11 in case 2. The average time to surgery for pelvic/acetabular fractures in our unit is 10 days, comparing favourably with other units around the country. It is possible that the bladder injury had become walled off with fibrous adhesions to the overlying rectus and was disturbed by the surgical approach. Although a trauma CT scan failed to detect a bladder rupture at day 1, no formal cystogram was performed in either case, and perhaps that could have identified the injury sooner.

Failure to diagnose a bladder rupture prior to pelvic stabilisation surgery has a number of implications. Not all hospitals in which pelvic surgery is undertaken have a urologist available at all times to assist with bladder repair. From a surgical planning perspective, ideally, the patient should be made aware of the possibility of a suprapubic catheter prior to surgery and the possible associated increased risk of metal-work infection. A medicolegal implication also exists in that a negative preoperative retrograde cystogram does not absolutely imply that a rupture found intraoperatively, or even postoperatively, was caused by the surgeon during fracture repair. Patients and surgeons should be aware that even contrast imaging of the bladder may not detect all injuries.
